# Downregulation of cAMP-Dependent Protein Kinase Inhibitor-b Promotes Preeclampsia by Decreasing Phosphorylated Akt

**DOI:** 10.1007/s43032-020-00258-8

**Published:** 2020-07-16

**Authors:** Chunfeng Liu, Hao Wang, Mo Yang, Yiheng Liang, Li Jiang, Siman Sun, Shangrong Fan

**Affiliations:** 1 Department of Obstetrics and Gynecology, Peking University Shenzhen Hospital, No. 1120 Lianhua Rd, Futian District, Shenzhen, 518036 China; 2 Shenzhen Key Laboratory on Technology for Early Diagnosis of Major Gynecological Diseases, Shenzhen, China; 3 Medical Center for Human Reproduction, Beijing Chaoyang Hospital, Capital Medical University, Beijing, China; 4 Center for Reproductive Medicine, Peking University Third Hospital, Beijing, China

**Keywords:** Preeclampsia, PKIB, Cell invasion, Cell migration, Akt pathway

## Abstract

Preeclampsia is a multi-system disease that is unique to human pregnancy. Impaired extravillous trophoblast migration and invasion accompanied by poor spiral vascular remodeling is thought to be the initial reason. This study investigated cAMP-dependent protein kinase inhibitor-b(PKIB) expression in placentas and its involvement in the pathogenesis of PE. We used immunohistochemistry and western blotting to calculate PKIB levels in the placentas. Then we knocked down PKIB by siRNA and used real-time cell analysis to assess the invasion and migration ability of trophoblasts. Tube formation assay and spheroid sprouting assay were utilized to identify the ability to form vessels of trophoblasts. At last, western blotting was used to demonstrate the level of phosphorylated Akt, as well as downstream-related genes of Akt signaling pathway in trophoblasts. We first found that PKIB expression level was lower in the PE placentas than in the normal placentas. In addition, we found that downregulation of PKIB can inhibit the migration, invasion, and the ability to form vessels of HTR8/SVneo cells. Downregulation of PKIB leaded to a decrease in phosphorylated Akt, as well as downstream proteins such as matrix metalloproteinase 2, matrix metalloproteinase 9, and glycogen synthase kinase 3β, which are related to migration and invasion. Our study revealed that the downregulation of PKIB expression resulted in decreased migration, invasion, and vessel formation ability by regulating Akt signaling pathway in placental trophoblasts in PE.

## Introduction

Preeclampsia (PE) is a pregnancy-specific clinical syndrome, with hypertension and proteinuria after 20 weeks of gestation as the major clinical manifestation [1]. PE affects an estimated 2–8% of pregnancies and is a global public health burden [2]. In China, the stillbirth rate is approximately 2.2% in women with PE [3]. Therefore, it is extremely important to understand PE pathogenesis in order to achieve early diagnosis and treatment. PE is thought to be a two-stage disorder: abnormal placental implantation in early pregnancy, followed by maternal inflammatory response [4, 5]. Until now, the accurate etiology has remained elusive, although the crucial involvement of extravillous trophoblasts (EVTs) is known [6]. During placental implantation of a successful pregnancy, the EVTs penetrate the endometrium and the underlying myometrium to induce intimate interactions between the placenta and uterine wall [7]. In PE, the EVTs migration and invasion ability is impaired, followed by poor spiral vascular remodeling and decreased volume of maternal blood flowing into the utero-placenta. This causes several negative consequences, including high pressure, abnormal stress in the villous placenta, increased shedding of necrotic placental debris into maternal vessels, and disruption of endothelium function [8–10]. However, the specific molecular mechanism of trophoblastic abnormalities leading to PE remains unclear.

cAMP-dependent protein kinase inhibitor-b (PKIB), a member of the protein kinase inhibitors (PKIs), is highly expressed in placental trophoblast cell [11]. Chung et al. observed that overexpression of PKIB in prostate cancer cells enhanced significantly the phosphorylation of Akt, a serine/threonine kinase, and promoted cell growth and invasion [12]. Besides, several other cancer studies have shown that PKIB overexpression promoted the occurrence and development of tumor through the Akt pathway, such as non-small cell lung cancer [13] and breast cancer [14, 15]. Akt is the main node in many signaling pathways and has a wide range of downstream substrates, such as MMP2, MMP9, GSK3β, procaspase-9, Bim, Bad, p21CIP1, and p27KIP1 [16–19]. Akt is activated through the phosphatidylinositol (3,4,5)-phosphate (PIP3)-PI3K pathway, and affects downstream targets that induce proliferation and cell cycle progression, apoptosis, migration, invasion, metabolism, angiogenesis, and metastasis [20, 21]. Trophoblast cells and cancer cells share many similarities in cell migration and invasion [22]. However, the effect of PKIB on trophoblast cells and PE is not clear.

In the current study, we examined the expression of PKIB in various placental trophoblasts and evaluated the effects of PKIB knockout in the HTR8/SVneo cell line. We also tested the hypothesis that PKIB could be a regulatory factor in the Akt pathway.

## Methods

### Collection of Placental Tissues

Placental tissues were collected from normal pregnancies (*n* = 15) and PE patients (*n* = 15) immediately after delivery who were admitted at the Peking University Shenzhen Hospital. PE was diagnosed by two or more times after the 20th week of gestation onset of systolic pressure ≥ 140 mmHg or diastolic blood pressure ≥ 90 mmHg and accompanied with the presence of proteinuria (at least 1+) and in the absence of preexisting renal diseases or primary hypertension [23]. Placental tissue of normal pregnancies and PE patients was both obtained by cesarean delivery. The gestational age of the PE group is from 33 + 4 to 39 + 4; premature placenta samples were iatrogenic preterm delivery (IPD) due to poor blood pressure control after clinical treatment. The gestational age of the control group is from 35 + 3 to 39 + 2 weeks. All women are cesarean section due to abnormal fetal position, cesarean history, fetal distress, cephalopelvic disproportion (CPD), or without indication. There were premature placenta samples in the control group because of early entering into the labor process with the problem of cesarean history; there was fetal distress because of the umbilical cord or abnormal fetal position like breech presentation, so cesarean section was used. All cases in the control and PE groups were excluded other placental abnormalities, including placenta previa and placental abruption; and without any other complications and comorbidities such as diabetes and heart diseases. A portion of each biopsy was immediately frozen in liquid nitrogen and stored at − 80 °C for further use, and the remaining tissue was fixed in 4% paraformaldehyde and then embedded in paraffin. All placental tissues were collected according to protocols approved by the Research Ethics Committee of Peking University Shenzhen Hospital, and written informed consent was obtained from all enrolled patients. The clinical features of the patients are displayed in Table 1.

**Table 1:** Clinical characteristics of participants

Category	Control (*n* = 15)	Preeclampsia (*n* = 15)	*p* value
Patient age (year)	29.11 ± 4.70	32.55 ± 3.58	0.08
Gestational age (weeks)	37.34 ± 0.67	36.88 ± 2.03	0.052
BMI at delivery (kg/m^2^)	27.78 ± 5.16	30.81 ± 3.87	0.151
Proteinuria (g/24 h)	–	2.89 ± 1.24	–
Systolic blood pressure (mmHg)	115.67 ± 15.6	153.09 ± 12.02	< 0.001
Diastolic blood pressure (mmHg)	75.11 ± 5.98	97.36 ± 9.18	< 0.001
Neonatal birth weight (g)	3327.78 ± 501.32	2580.45 ± 795.13	0.020
Nulliparity	7(46.67%)	6(40.0%)	0.713

All placentas were obtained from elective, non-labored cesarean deliveries

*BMI*, body mass index (kg/m^2^); *BP*, blood pressure. Values are mean ± standard deviation or *n*(%).

### Immunohistochemistry (IHC)

IHC was used to detect the expression of PKIB in placentas. The placental tissues and villous were washed with phosphate-buffered saline (PBS) and fixed with 4% paraformaldehyde (PFA) at room temperature overnight. Then, the samples were dehydrated, embedded in paraffin, and sected into 4-um-thick sections. Then the sections were deparaffinized, rehydrated, and then microwaved in 10 mM citric sodium (pH 6.0) for 20 min to retrieve antigens and blocked with 3% H_2_O_2_ for 10 min. The sections were then incubated with a mouse primary antibody against PKIB (1:200; Immunoway, Suzhou, China) at 4 °C overnight. The next day, applied secondary antibody conjugated to horseradish peroxidase (HRP) for 20 min, followed by diaminobenzidine solution development.

### RNA Extraction and Real-Time Quantitative PCR

RNA was extracted using Trizol RNA Isolation Reagents (Thermo Fisher Scientific) according to the manufacturer’s instructions. Reverse transcription was performed using 1 mg of total RNA, which was converted into cDNA using a RevertAid First Strand cDNA Synthesis Kit (Thermo Fisher Scientific) following the manufacturer’s instructions. Real-time PCR was performed using SYBR Green (Thermo Fisher Scientific) in a QuantStudio 3 Real-Time PCR System (Thermo Fisher Scientific). Transcripts were quantified from the corresponding standard curve using *β-actin* as an internal control. Each sample was run in triplicate, and each experiment was performed three times. The following primers were used: forward 5′-GGCACATACTAGAAGCAAAATACG-3′ and reverse 5′-GATGGGCAAATCATTCTTGGTA-3′ for *PKIB*; forward 5′-TTGGCTTGACTCAGGATTTA-3′ and reverse 5′-ATGCTATCACCTCCCCTGTG-3′ for *β-actin* (*ACTB*) [12].

### Western Blot (WB) Assay

Protein extracts were prepared from HTR8/SVneo cell line and placental tissues with RIPA buffer supplemented with protease inhibitors. For WB analysis, the cell lysates (20 μg of total protein) were electrophoresed by 10% SDS-PAGE and electrically transferred to a hydrophobic polyvinylidene difluoride membrane (Hybond-P; Amersham Biosciences, Piscataway, NJ, USA). Following transfer, the membranes were blocked in 5% milk in TBST (0.15 M NaCl, 0.01 M Tris-HCl, and 0.1% Tween-20, pH 7.4) for 1 h, and then the membranes were incubated with primary antibodies (PKIB 1:200, Immunoway, Suzhou, China; β-actin 1:1000, CST, Danvers, MA, USA; AKT 1:1000, CST; Phospho-Akt(Ser473) 1:1000, CST; MMP2 1:1000, CST; MMP9 1:1000, CST; GSK3β 1:1000, CST) overnight at 4  °C. After using TBST to wash three times, the membrane was incubated with an appropriate secondary antibody for 1 h. Then the membranes were detected with an enhanced chemiluminescence detection system (Amersham Biosciences, Piscataway, NJ, USA).

### Cell Culture

HTR8/SVneo cell line (ATCC No. CRL-3271) is an immortalized cell line formed by transfecting the large T antigen of SV40 virus into human primary placenta cells in early pregnancy. HTR8/Svneo’s biological functions and traits are similar to primary cells, and is suitable for PE research. Cells were cultured in phenol red RPMI 1640 medium (Thermo Fisher Scientific, Waltham, MA, USA). Cells were grown at 37 °C in 5% CO_2_. All cultures were supplemented with 10% fetal bovine serum (FBS, Thermo Fisher Scientific) and 100 U/mlpenicillin-streptomycin (Thermo Fisher Scientific).

### Small Interfering RNA Design and Transfection

Small interfering RNAs (siRNAs) that target the PKIB gene and the negative control siRNA, which does not target any sequence present in PKIB or the human genome, were purchased from Shanghai GenePharma (Shanghai, China). Lipofectamine 3000 Transfection Reagent (Thermo Fisher Scientific) was used to transiently transfect the cells with siRNA according to the manufacturer’s instructions. PKIB siRNA sequence: 5′-GCAGTAGGC ACTTAAGCAT-3′ and control siRNA sequence: 5′-GCGCGCTTTGTAGGATTCG-3′ [12].

### Real-Time Cell Migration and Invasion Assays

We used real-time cell analysis (RTCA) system (RTCA DP Instrument; ACEA Biosciences, Inc., USA) to examine cell migration and invasion abilities. To monitor cell migration continuously, the cells were seeded at a density of 2 × 10^4^ cells per well in RTCA Cell Invasion Migration (CIM)-16 plates, with each well consisting of an upper and a lower chamber separated by a microporous membrane that could detect migrating cells, and then the electrical impedance in each well was measured continuously for 80 h. The shift in electrical impedance is expressed as the cell index, which is a parameter of cell migration. For the invasion assay, a layer of Matrigel was added to the upper chamber of the CIM plates for an hour to simulate extracellular matrix (ECM) and provide a barrier for cell invasion [24]. Next, 2 × 10^4^ cells were seeded into each well and monitored for 80 h. The sensor impedance following cell invasion was defined as the cell index.

### Tube Formation Assay

Matrigel was placed in a 96-well cell culture plate (50 μl/well) and incubated for 30 min. 3 × 10^4^HTR8/SVneo cells (100 μl) were seeded onto Matrigel-coated wells, and cultured for 6 h until tube formation was observed. Then, the medium was aspirated from the well, and 100 μl of 2 μM Calcein AM (Abcam) solution was added to each well, followed by incubation of the plate for 20 min. Tube formation was observed under an inverted microscope. Calcein AM-labeled cells were photographed using a fluorescence inverted microscope at an excitation wavelength of 488 nm.

### Spheroid Sprouting Assay

Spheroids were generated as previously reported [25]. Briefly, cells resuspended in endothelial basal medium (EBM) containing 0.24% high viscosity methylcellulose (Sigma-Aldrich), and were seeded in 96-well round bottom non-adherent plates. Cells were incubated overnight at 37 °C. Each spheroid contained 1 × 10^3^ cells. Individual spheroids were harvested, embedded in rat tail collagen type 1 gel (Corning, Seneffe, Belgium), supplemented with EBM, and incubated for 48 h at 37 °C. To quantify germination, the average number of shoots under each condition was calculated.

### Statistical Analyses

The unpaired Student’s *t* test was used for continuous variables using GraphPad Prism 6 software (La Jolla, CA, USA), and independence between categorical variables were assessed with Chi-square tests. Data are represented as means and standard deviations or *n*(%). All experiments were performed three times unless indicated otherwise. A *p* value < 0.05 was considered to indicate a statistically significant difference.

## Results

### Expression of PKIB in Placentas

**Fig. 1:**
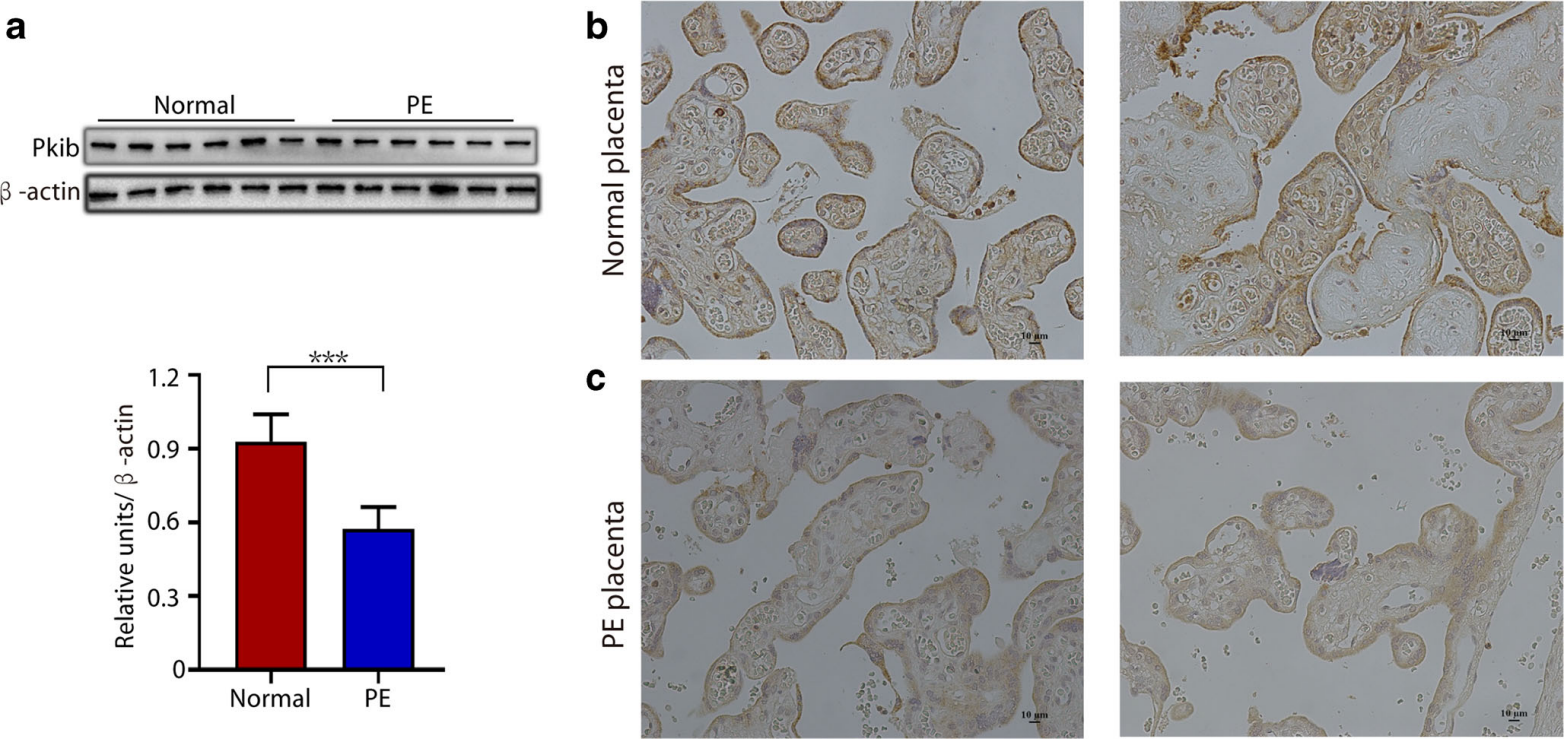
PKIB expression in placentas. **a** The expression of PKIB in placental tissues was determined by western blot analysis in preeclampsia (PE) placentas and normal control placentas. Data are presented as mean ± standard error of the mean. ***p < 0.001. **b**, **c** IHC staining of PKIB of normal and PE placentas on serial sections. Scale bar, 10 μm

First, we performed WB assays to compare the differences in PKIB expression between PE and normal gestational placentas. As shown in Table 1, there were no statistical differences in maternal ages, gestational ages, and body mass index between normal pregnant and PE women. WB results demonstrated that the expression level of PKIB was significantly lower in PE placentas than in normal placentas (Fig. 1a). In accordance with this, IHC staining results further revealed consistently high PKIB staining in the trophoblast of the basal plate of normal placentas, whereas PKIB was weakly expressed in PE placentas (Fig. 1b, c).

### PKIB Regulates Trophoblast Migration and Invasion

**Fig. 2:**
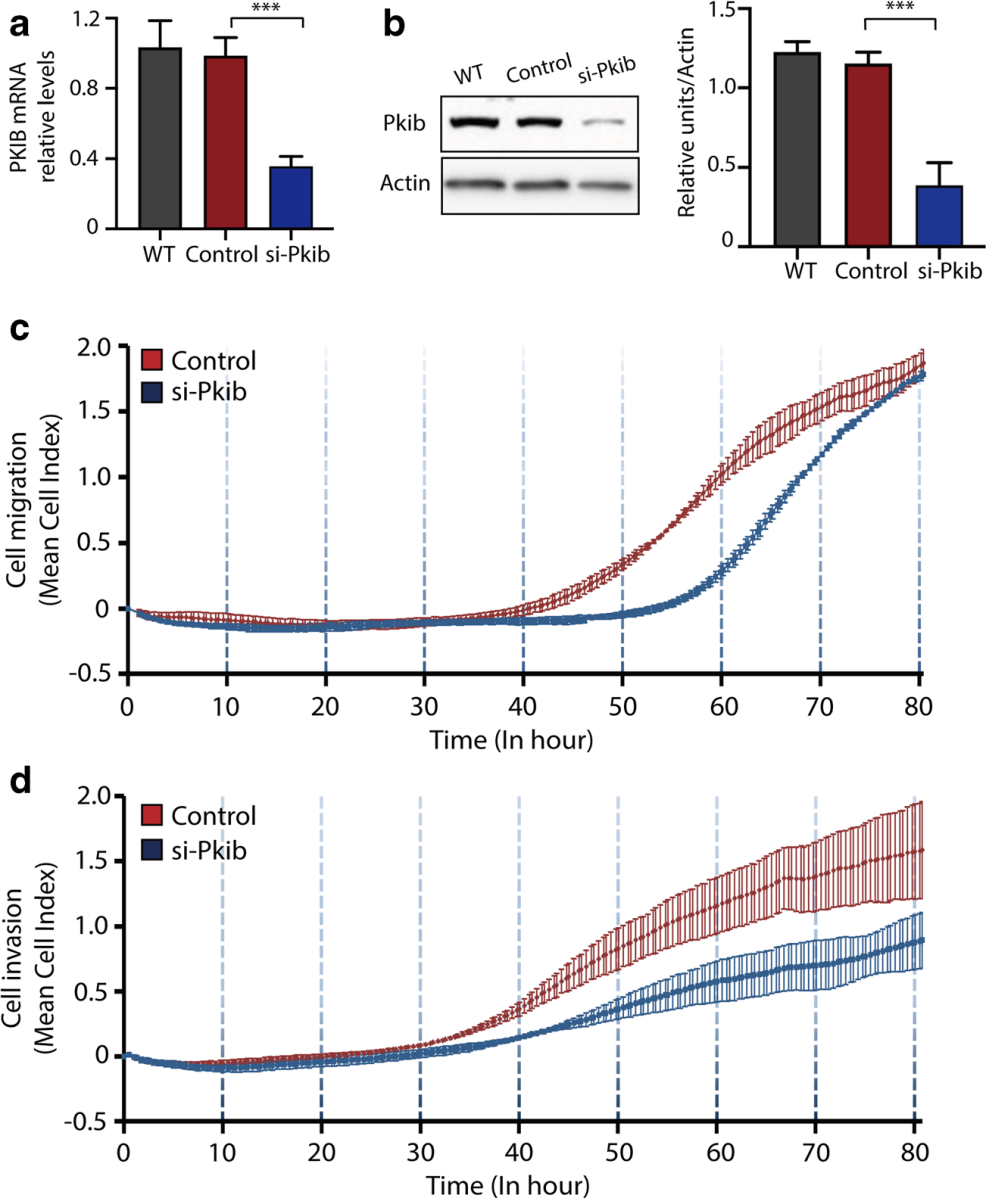
PKIB regulates trophoblast migration and invasion. **a** Validation of PKIB knockdown (PKIB-KD) by real-time PCR. **b** Validation of PKIB-KD by western blot analysis. Equal amounts of normal HTR8/Svneo (control) and PKIB cell lysates were subjected to western blot analysis with a PKIB antibody. **c** Migration cell Index (CI) based on real-time cell analysis (RTCA) system. **d** Invasion CI based on RTCA system. All experiments were performed at least three times. WT, wild type trophoblast; Control, cells with scrambled-siRNA; siPKIB, cells with target siRNA. Values are represented as mean ± standard error of mean. **p < 0.01, ***p < 0.001

We first established a cell line in which PKIB was knocked down using siRNA in HTR8/SVneo, an EVT-like first trimester trophoblast cell line. Real-time qPCR and WB results confirmed successful knockdown of PKIB in the cell with target si-RNA, while the scrambled-siRNA cells and wild type (WT) trophoblasts are nearly the same, so we used scrambled-siRNA cells as the control (Fig. 2a, b). Then, we used RTCA to explore the function of PKIB in cell migration and invasion. Transfected si-PKIB and si-control cells were seeded into CIM-plates. When the cells passed through the membrane from the upper chamber into the bottom, they contacted the sensors at the membrane, which was measured as the cell index. After 80 h of culturing, the cell migration ability was significantly decreased in the si-PKIB group compared with that in the control group (Fig. 2c). And as shown in Fig. 2d, the invasion ability of cells in the si-PKIB group was also much lower than that in the control group.

### PKIB Regulates Tube Formation in Trophoblasts

**Fig. 3:**
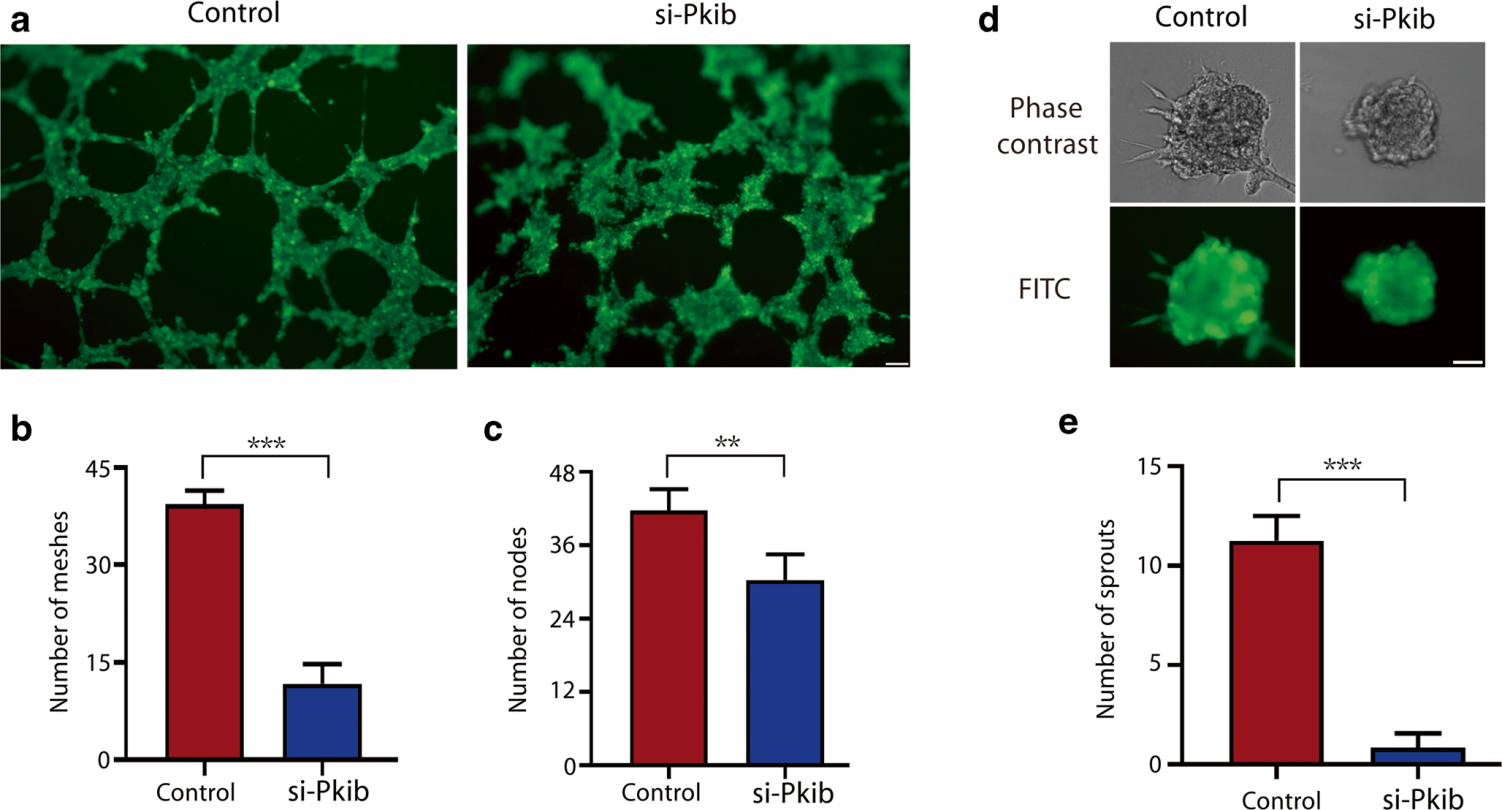
PKIB regulates tube formation in trophoblasts. **a** Matrigel endothelial-like tube formation was examined in HTR8/Svneo cells. Scale bar, 100 μm. **b** Number of meshes. **c** Number of nodes. **d** Spheroid sprouting assay was examined in HTR8/Svneo cells. **e** Number of sprouts. All experiments were performed at least three times. All data are represented as the mean ± standard error of mean. **p < 0.01, ***p < 0.001

As shown in Fig. 3a, the si-PKIB cells exhibited decreased ability of tube formation compared with the control cells. The results of quantitative analysis demonstrated that the numbers of nodes and meshes were significantly reduced (Fig. 3b, c). Furthermore, spheroid sprouting assay confirmed that angiogenic properties of trophoblasts were decreased by knockdown of PKIB (Fig. 3d, e).

### Effects of PKIB Are Mediated by Regulating Phosphorylated Akt Expression

**Fig. 4:**
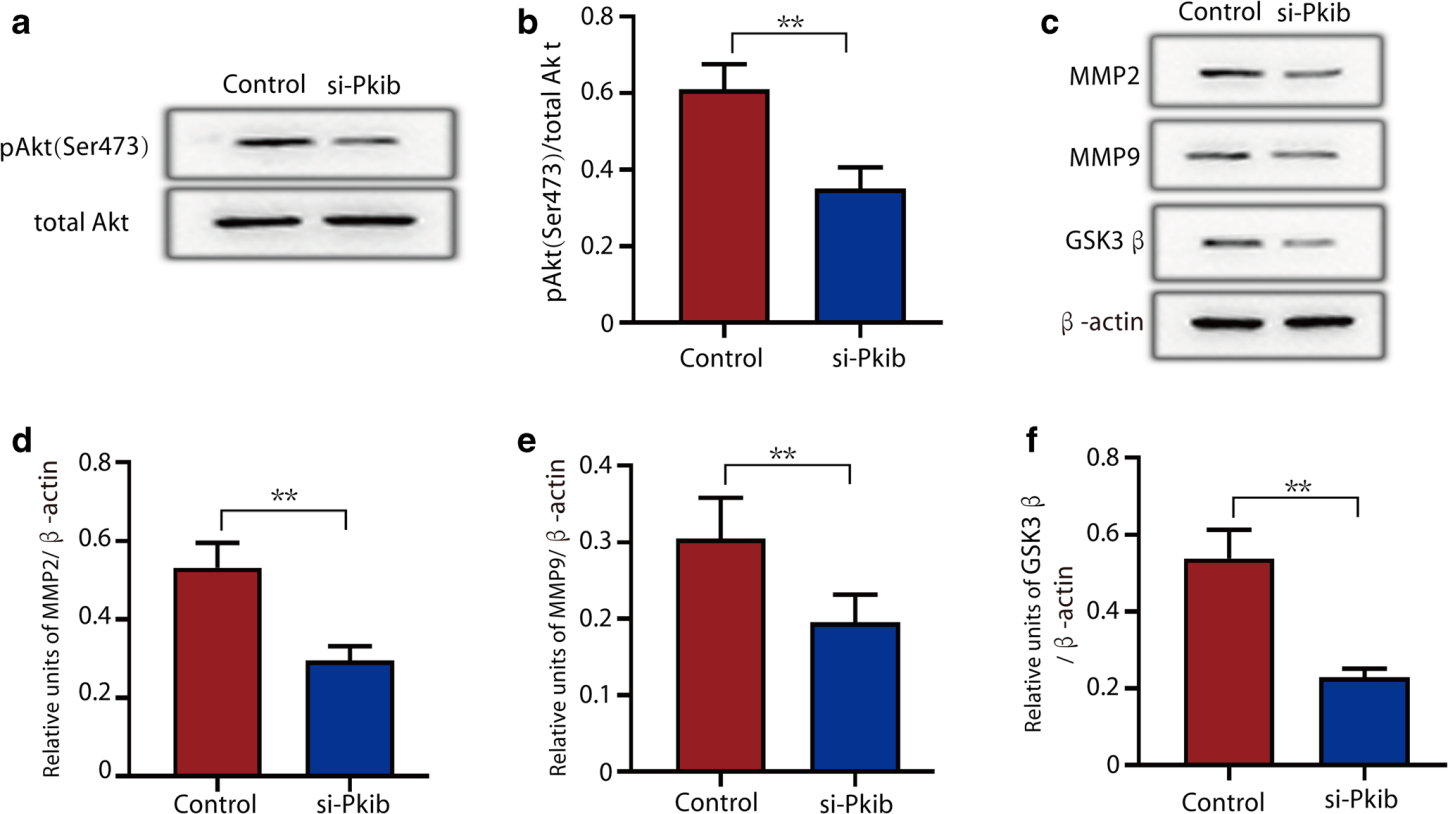
PKIB promotes trophoblast proliferation and invasion by activating the Akt pathway. **a** Expression of phosphorylated Akt-Ser473 and total Akt in transfected si-control and si-PKIB was determined by western blot analysis. **b** Relative intensities of bands are shown in the histogram. **c** Expression of MMP2, MMP9, and GSK3β in transfected si-control and si-PKIB was determined by western blot analysis. β-actin was used as a loading control. **d**–**f** Relative intensities of **d** MMP2, **e** MMP9, and **f** GSK3β to β-actin. All data are represented as the mean ± standard error of mean. Error bars represent the standard deviation. **p < 0.01, ***p < 0.001

WB analysis indicated that downregulation of PKIB resulted in decreased Akt phosphorylation of Ser473. (Fig. 4a, b), while the total amount of Akt was not significantly affected. Silencing PKIB also decreased the expression of Akt downstream genes. Using WB, we found that MMP-2 and MMP-9 decreased significantly in HTR8/SVneo cells in response to PKIB knockdown (Fig. 4c, d).

## Discussion

PE is a pregnancy-specific disease, and the cause is still elusive. Placental delivery is the only effective treatment for PE [26]. Trophoblasts are important in placental development, and migration and invasion of trophoblasts are strictly controlled during pregnancy [27]. Defective trophoblast invasion with incomplete vascular transformation and inadequate uteroplacental perfusion may contribute to PE and other pregnancy-related diseases [28]. Although migration and invasiveness are common characteristics between trophoblasts and carcinomas, unlike cancer cells, the trophoblastic invasion during placentation is stringently controlled in both space and time [29], but the specific molecular mechanism of regulation is still unclear. We investigated that PKIB participates in PE development by promoting trophoblast migration and invasion. In this study, we found trophoblasts of PE placentas showed significantly decreased protein expression, compared to those of control placentas. In addition, migration and invasion abilities were inhibited significantly in the PKIB knockdown cells, indicating the regulatory role of PKIB on trophoblast function.

Limited remodeling of the spiral arteries is also an important part of PE pathogenesis. It has been reported that late sporadic abortion and PE may be associated with decreased endometrial spiral invasion and insufficient intermuscular spiral arterial transition [30]. The narrow spiral arteries are prone to atherosis, which are characterized by perivascular mononuclear inflammatory infiltrates, lipid-rich macrophages within vessel lumens, and fibrin-like necrosis of the wall invasions, resulting to further uteroplacental ischemia [31, 32]. Then, the interstitial space forms a vicious circle of ischemia and reperfusion, aggravates secondary thrombotic lesions, limits blood entering the placenta, and causes infarction [33]. Therefore, we also explored the effect of PKIB on angiogenesis. When the HTR8/SVneo cell line is cultured on Matrigel (with or without endothelial cells), it shows intrinsic ability to spontaneously form endothelial-like tubes, which is believed to reflect the ability of placental angiogenesis [34, 35]. The total lengths of the tubular network and branches were measured in pixels. Through tube-forming experiments and 3D cell culture, we demonstrated knockdown of PKIB in trophoblast could impede angiogenesis.

Given that low expression of PKIB plays an important role in PE process, the underlying mechanisms of these functions require further investigation. Several signaling pathways, including the PI3K/Akt signaling pathway, play key roles in cell growth and survival under physiological and pathological conditions, particularly cell migration and invasion [36]. A large number of reports indicated that PKIB was a functional partner of Akt and played an important role especially in Akt phosphorylation and signal transduction of tumor cells [14, 15]. We found that downregulation of PKIB was related to significantly downregulated levels of p-Akt in HTR8/SVneo cells. This may indicate that PKIB exerts its effects by regulating the expression of phosphorylated Akt. We next investigated the effect of PKIB on Akt downstream gene expression in HTR8/SVneo cells, including MMP-2, MMP-9, and GSK3β, which are closely related to cell migration and invasion [37–39], and found that reduced expression of MMP2, MMP9, and GSK3β in the si-PKIB group. Based on this, we speculated that the effects of PKIB on trophoblast migration and invasion likely occur via the Akt pathway.

In summary, our study showed that PKIB is mainly expressed in placental trophoblasts, and compromised in PE-complicated pregnancies. Downregulation of PKIB suppresses trophoblast migration, invasion, and angiogenesis through inhibiting Akt phosphorylation. Our novel insights into role of PKIB will be useful for understanding mechanism of PE.

## References

[1] Mol BW, Roberts CT, Thangaratinam S, Magee LA, De Groot CJ, Hofmeyr GJ (2016). Pre-eclampsia. Lancet.

[2] Abalos E, Cuesta C, Carroli G, Qureshi Z, Widmer M, Vogel J, Souza JP, on behalf of the WHO Multicountry Survey on Maternal and Newborn Health Research Network (2014). Pre-eclampsia, eclampsia and adverse maternal and perinatal outcomes: a secondary analysis of the W orld H ealth O rganization multicountry S urvey on M aternal and N ewborn H ealth. BJOG Int J Obstet Gynaecol.

[3] Xiong T, Mu Y, Liang J, Zhu J, Li X, Li J, Liu Z, Qu Y, Wang Y, Mu D (2018). Hypertensive disorders in pregnancy and stillbirth rates: a facility-based study in China. Bull World Health Organ.

[4] Roberts JM, Hubel CA (2009). The two stage model of preeclampsia: variations on the theme. Placenta..

[5] Redman CW, Staff AC (2015). Preeclampsia, biomarkers, syncytiotrophoblast stress, and placental capacity. Am J Obstet Gynecol.

[6] DaSilva-Arnold S, James JL, Al-Khan A, Zamudio S, Illsley NP (2015). Differentiation of first trimester cytotrophoblast to extravillous trophoblast involves an epithelial–mesenchymal transition. Placenta.

[7] Pijnenborg R, Bland J, Wa R, Brosens I (1983). Uteroplacental arterial changes related to interstitial trophoblast migration in early human pregnancy. Placenta.

[8] Burton G, Yung H-W, Cindrova-Davies T, Charnock-Jones D (2009). Placental endoplasmic reticulum stress and oxidative stress in the pathophysiology of unexplained intrauterine growth restriction and early onset preeclampsia. Placenta.

[9] Redman CW, Sargent IL (2005). Latest advances in understanding preeclampsia. Science.

[10] Harris LK, Benagiano M, D’Elios MM, Brosens I, Benagiano G. Placental bed research: II. Functional and immunological investigations of the placental bed. Am J Obstet Gynecol. 2019;221(5):457–69. 10.1016/j.ajog.2019.07.010 31288009

[11] Zheng L, Long Y, Qiang T, Zhang M, Hua H, Wengjie C (2000). Cloning and mapping of human PKIB and PKIG, and comparison of tissue expression patterns of three members of the protein kinase inhibitor family, including PKIA. Biochem J.

[12] Chung S, Furihata M, Tamura K, Uemura M, Daigo Y, Nasu Y, Miki T, Shuin T, Fujioka T, Nakamura Y, Nakagawa H (2009). Overexpressing PKIB in prostate cancer promotes its aggressiveness by linking between PKA and Akt pathways. Oncogene.

[13] Dou P, Zhang D, Cheng Z, Zhou G, Zhang L (2016). PKIB promotes cell proliferation and the invasion-metastasis cascade through the PI3K/Akt pathway in NSCLC cells. Exp Biol Med.

[14] Dabanaka K, Chung S, Nakagawa H, Nakamura Y, Okabayashi T, Sugimoto T, Hanazaki K, Furihata M (2012). PKIB expression strongly correlated with phosphorylated Akt expression in breast cancers and also with triple-negative breast cancer subtype. Med Mol Morphol.

[15] Zhang J, Song W, Wang Y, Liu M, Sun M, Liu H (2017). Study on correlation between PKIB and pAkt expression in breast cancer tissues. Eur Rev Med Pharmacol Sci.

[16] McCubrey JA, Steelman LS, Chappell WH, Abrams SL, Wong EW, Chang F (2007). Roles of the Raf/MEK/ERK pathway in cell growth, malignant transformation and drug resistance. Biochim Biophys Acta.

[17] Staun-Ram E, Goldman S, Gabarin D, Shalev E (2004). Expression and importance of matrix metalloproteinase 2 and 9 (MMP-2 and-9) in human trophoblast invasion. Reprod Biol Endocrinol.

[18] Tran H, Brunet A, Griffith EC, Greenberg ME (2003). The many forks in FOXO’s road. Sci STKE.

[19] Qi X-J, Wildey GM, Howe PH (2006). Evidence that Ser87 of BimEL is phosphorylated by Akt and regulates BimEL apoptotic function. J Biol Chem.

[20] Mundi PS, Sachdev J, McCourt C, Kalinsky K (2016). AKT in cancer: new molecular insights and advances in drug development. Br J Clin Pharmacol.

[21] Engelman JA (2009). Targeting PI3K signalling in cancer: opportunities, challenges and limitations. Nat Rev Cancer.

[22] Ferretti C, Bruni L, Dangles-Marie V, Pecking A, Bellet D (2007). Molecular circuits shared by placental and cancer cells, and their implications in the proliferative, invasive and migratory capacities of trophoblasts. Hum Reprod Update.

[23] Backes CH, Markham K, Moorehead P, Cordero L, Nankervis CA, Giannone PJ (2011). Maternal preeclampsia and neonatal outcomes. J Pregnancy.

[24] Limame R, Wouters A, Pauwels B, Fransen E, Peeters M, Lardon F, de Wever O, Pauwels P (2012). Comparative analysis of dynamic cell viability, migration and invasion assessments by novel real-time technology and classic endpoint assays. PLoS One.

[25] Amand M, Erpicum C, Bajou K, Cerignoli F, Blacher S, Martin M, Dequiedt F, Drion P, Singh P, Zurashvili T, Vandereyken M, Musumeci L, Mustelin T, Moutschen M, Gilles C, Noel A, Rahmouni S (2014). DUSP3/VHR is a pro-angiogenic atypical dual-specificity phosphatase. Mol Cancer.

[26] Phipps E, Prasanna D, Brima W, Jim B (2016). Preeclampsia: updates in pathogenesis, definitions, and guidelines. Clin J Am Soc Nephrol.

[27] Red-Horse K, Zhou Y, Genbacev O, Prakobphol A, Foulk R, McMaster M, Fisher SJ (2004). Trophoblast differentiation during embryo implantation and formation of the maternal-fetal interface. J Clin Invest.

[28] Norwitz ER (2006). Defective implantation and placentation: laying the blueprint for pregnancy complications. Reprod BioMed Online.

[29] Bischof P, Meisser A, Campana A (2000). Mechanisms of endometrial control of trophoblast invasion. J Reprod Fertil Suppl.

[30] Ball E, Bulmer J, Ayis S, Lyall F, Robson S (2006). Late sporadic miscarriage is associated with abnormalities in spiral artery transformation and trophoblast invasion. J Pathol.

[31] Mayrink J, Costa M, Cecatti J (2018). Preeclampsia in 2018: revisiting concepts, physiopathology, and prediction. Sci World J.

[32] Rana S, Lemoine E, Granger JP, Karumanchi SA (2019). Preeclampsia: pathophysiology, challenges, and perspectives. Circ Res.

[33] Wang A, Rana S, Karumanchi SA (2009). Preeclampsia: the role of angiogenic factors in its pathogenesis. Physiology.

[34] Labarrere CA, DiCarlo HL, Bammerlin E, Hardin JW, Kim YM, Chaemsaithong P (2017). Failure of physiologic transformation of spiral arteries, endothelial and trophoblast cell activation, and acute atherosis in the basal plate of the placenta. Am J Obstet Gynecol.

[35] Basak S, Duttaroy AK (2013). cis-9, trans-11 conjugated linoleic acid stimulates expression of angiopoietin like-4 in the placental extravillous trophoblast cells. Biochim Biophys Acta.

[36] Alzahrani AS, editor. PI3K/Akt/mTOR inhibitors in cancer: at the bench and bedside Seminars in cancer biology; 2019: Elsevier. 10.1016/j.semcancer.2019.07.009 31323288

[37] Wang R, Wang W, Ao L, Wang Z, Hao X, Zhang H (2017). Benzo [a] pyrene-7, 8-diol-9, 10-epoxide suppresses the migration and invasion of human extravillous trophoblast HTR-8/SVneo cells by down-regulating MMP2 through inhibition of FAK/SRC/PI3K/AKT pathway. Toxicology.

[38] Liang X, Sun R, Zhao X, Zhang Y, Gu Q, Dong X, Zhang D, Sun J, Sun B (2017). Rictor regulates the vasculogenic mimicry of melanoma via the AKT-MMP-2/9 pathway. J Cell Mol Med.

[39] Embi N, Rylatt DB, Cohen P (1980). Glycogen synthase kinase-3 from rabbit skeletal muscle: separation from cyclic-AMP-dependent protein kinase and phosphorylase kinase. Eur J Biochem.

